# Magnetic Resonance Imaging Findings in a Rare Case of a Ruptured Intracranial Dermoid Cyst

**DOI:** 10.7759/cureus.43316

**Published:** 2023-08-10

**Authors:** Iram Saifi, Shivali V Kashikar, Rajasbala Dhande, Asish Pavanan, Sai Nidhi G Reddy

**Affiliations:** 1 Radiodiagnosis, Jawaharlal Nehru Medical College, Wardha, IND

**Keywords:** ruptured intracranial dermoid, intracranial dermoid and radiological findings, mri findings of a dermoid cysts, intracranial dermoid cyst location, dermoid cyst radiology, mri findings of dermoid, rupture of intracranial dermoid, intracranial dermoid cyst

## Abstract

Intracranial dermoid cysts are rare, benign, congenital, and slow-growing cystic lesions. They contain mature squamous epithelium, apocrine, eccrine, sebaceous glands, and ectodermal structures. The rupture of intracranial dermoid cysts is a rare event and can cause life-threatening conditions.

## Introduction

About 1% of all intracranial masses are dermoid cystic tumours. Ectodermal inclusion cysts, which are made up of several ectoderm derivatives such as apocrine, sweat, and sebaceous cysts, are present in these dermoid cysts, which are nonneoplastic and congenital. Additionally, they could have teeth, squamous epithelium, and hair follicles. The distinction between these dermoid cysts and epidermoid cysts must be made, as the latter only includes squamous epithelium [1].

Intracranial dermoid cysts are more prevalent in females and account for less than 0.5% of all primary cerebral tumours. Dermoid cysts typically appear within the first three decades of life. The midline suprasellar region is where they most frequently occur. Although extremely uncommon, they can transform into a malignancy like squamous cell carcinoma. Dermoid cysts typically do not cause symptoms unless they rupture. The rupture of these cysts is thought to occur due to the release of hair and oils from the internal dermal components, leading to an increase in pressure. It's worth noting that trauma can also be a factor preceding the rupture of dermoid cysts [2].

## Case presentation

**Figure 1:**
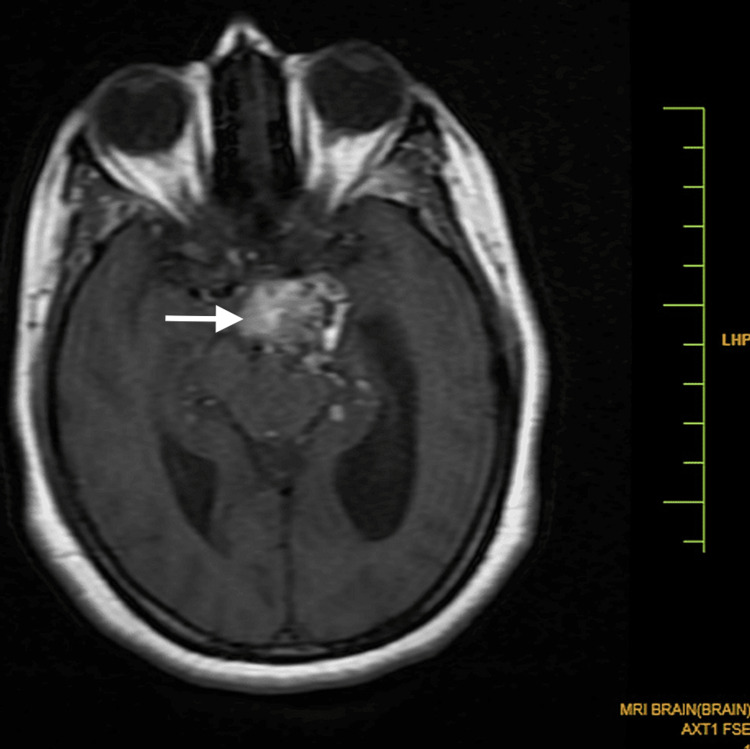
An axial MR T1WI shows a heterogenous lobulated lesion in the region of the left side of the interpedicular cistern extending to the prepontine cistern measuring 3 x 2.5 x 4 cm, predominantly hyperintense on T1WI with some hypointense areas within. Self-clicked image MR: magnetic resonance; T1WI: T1 weighted image

**Figure 2:**
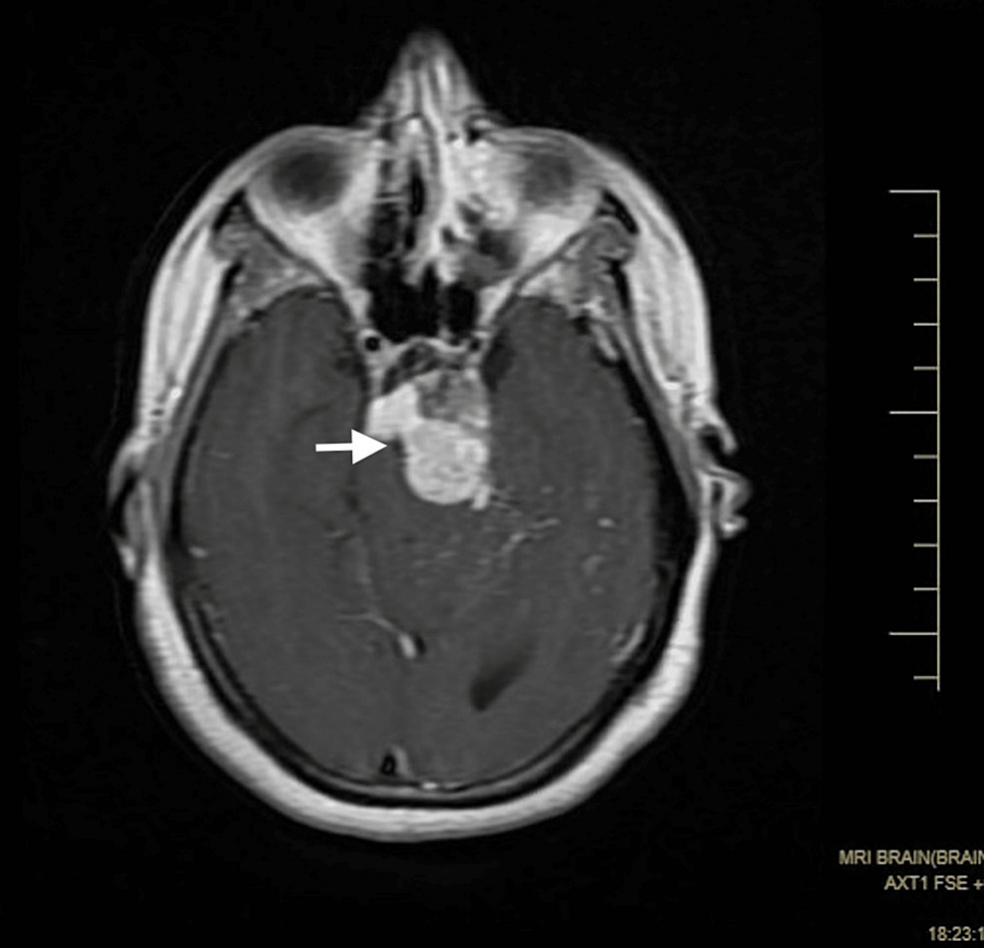
An axial MR T1WI (contrast) shows heterogeneous lobulated lesions in the region of the left side of the interpedicular cistern extending to the prepontine cistern. Self-clicked image MR: magnetic resonance; T1WI: T1 weighted image

**Figure 3:**
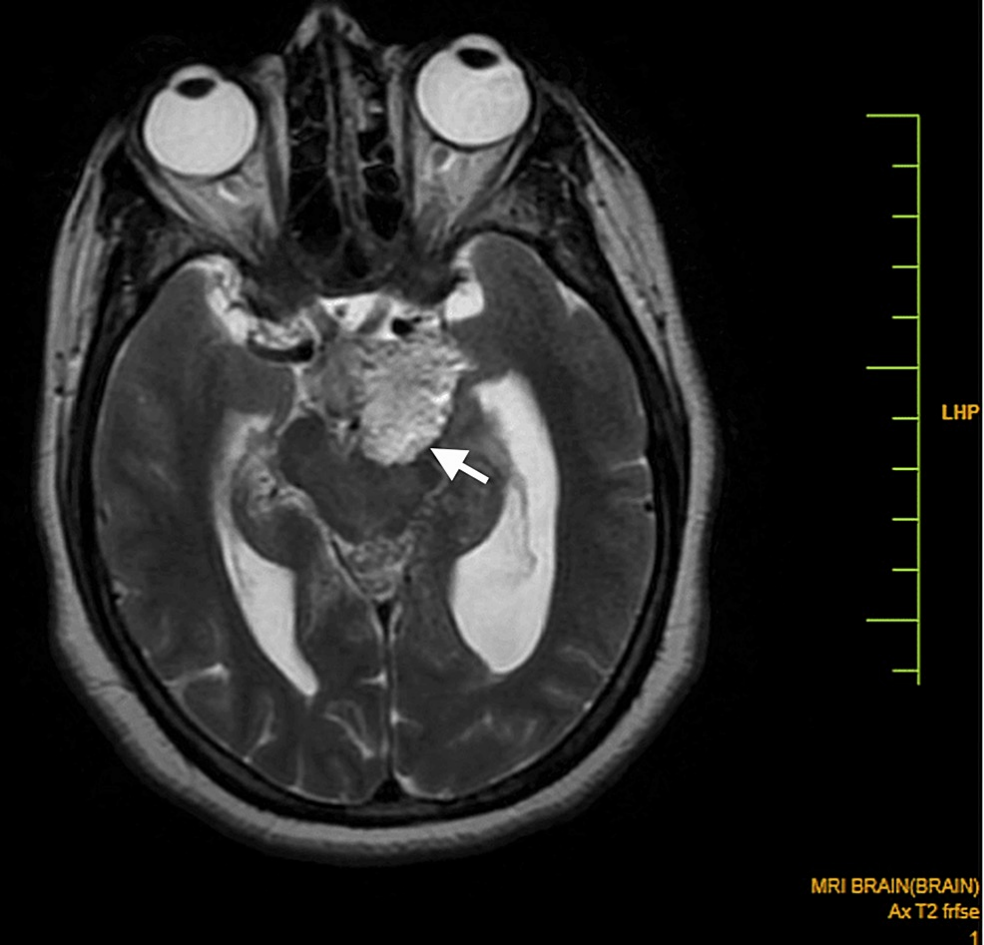
An axial MR T2WI shows a heterogeneous hyperintense lobulated lesion in the region of the left side of the interpedicular cistern extending to the prepontine cistern. Self-clicked image MR: magnetic resonance; T2WI: T2 weighted image

A 40-year-old woman came to the hospital with complaints of a decrease in left-sided vision, decreased palpebral fissures, and left-sided diplopia. She had intense, sharp, throbbing, sporadic, burning, and shock-like pain around the eyes, lips, nose, jaw, forehead, and scalp on the left side. Attacks of sharp pain were triggered by touch. She had a history of occasional headaches, too. Vital signs were within normal limits. On examination, the eyeball was downward and outward. Plain and contrast MRIs of the brain were requested, which revealed a heterogenous lobulated lesion in the region of the left side of the interpeduncular cistern extending to the prepontine cistern measuring 3 x 2.5 x 4 cm (Figures 1-3).

**Figure 4:**
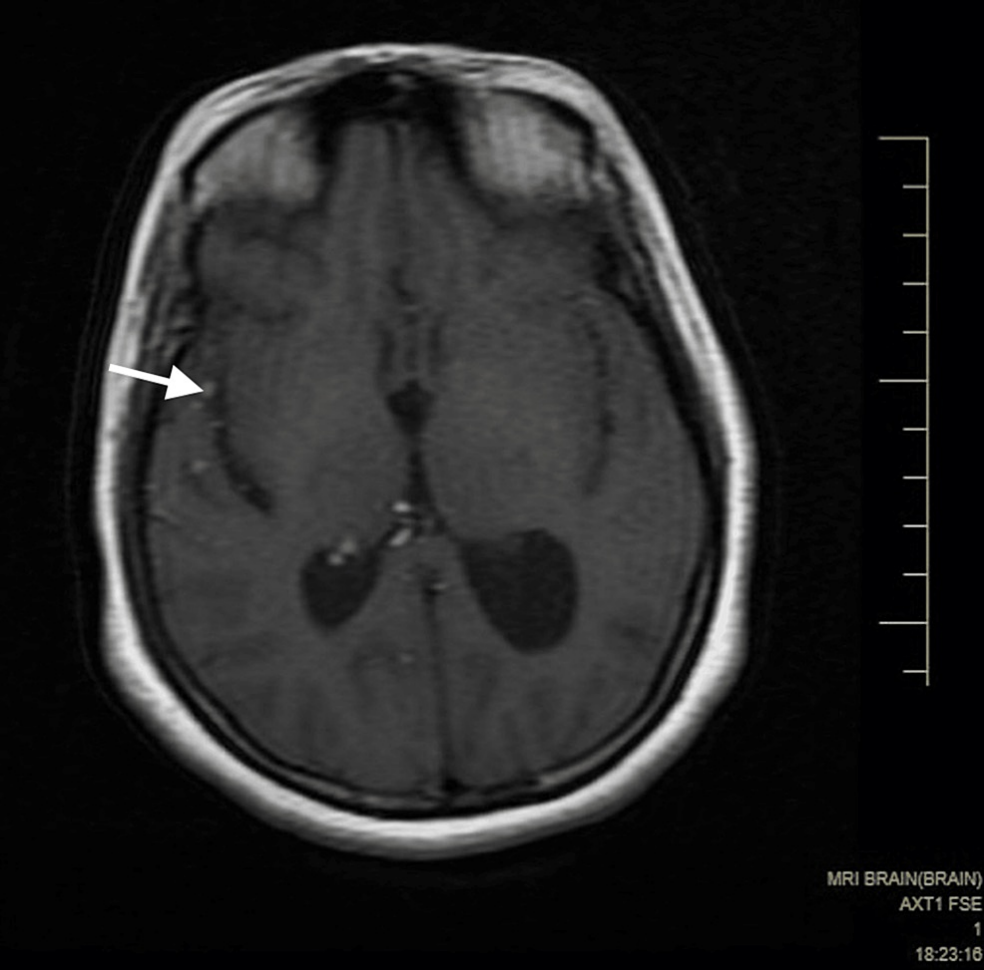
An axial MR T1WI shows multiple tiny T1 hyperintensities diffusely scattered in subarachnoid spaces in the brain, likely to be fat. Self-clicked image MR: magnetic resonance; T1WI: T1 weighted image

A small component extended into the left cistern anterolateral to the pons in the left cerebellopontine angle. It was predominantly hyperintense on both the T1 weighted image (T1WI) and the T2 weighted image (T2WI), with some hypointense areas within, without significant enhancement (Figure 1, 2). Tiny T1 hyperintensities were seen diffusely scattered in subarachnoid spaces in the brain, likely to be fat (Figure 4).

**Figure 5:**
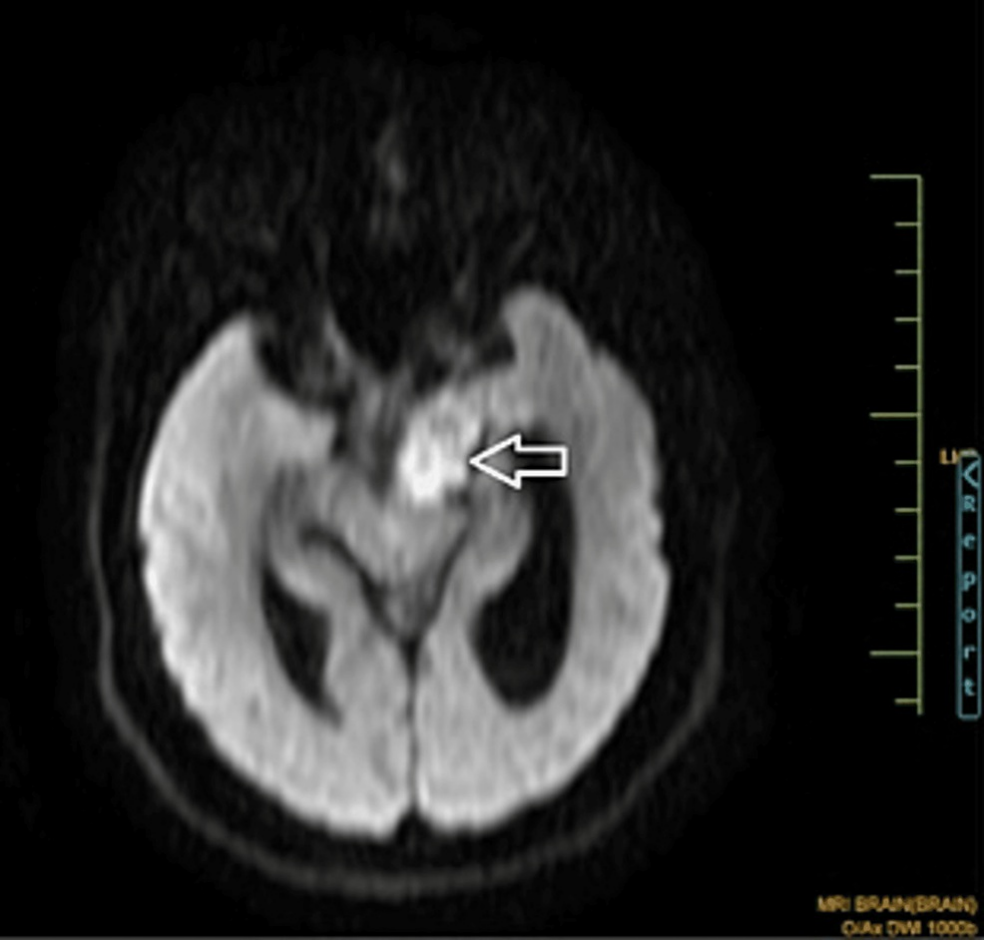
An axial MR DWI showing restricted diffusion Self-clicked image MR: magnetic resonance; DWI: diffusion-weighted imaging

There was a mass effect on the left mid-brain and the left pons. Anteriorly, it abutted the left internal carotid artery. Restricted diffusion was seen (Figure 5).

In our case, the patient went for a surgical approach and had a craniotomy with the removal of the dermoid cyst and irrigation of subarachnoid spaces to remove ruptured contents and debris.

## Discussion

Dermoid cysts usually develop near the base of the skull, with the sellar or parasellar region and the frontonasal region being popular sites. Numerous symptoms, including headaches, seizures, cerebral ischemia, and meningitis, can be brought on by these cysts. Dermoid cysts normally burst on their own; however, there have been a few instances of forced rupture. Dermoid cysts vary in their clinical presentation depending on where they are located. Headaches (32.6%) and seizures (26.5%), cerebral ischemia (16.3%), and aseptic meningitis (8.2%) are the most frequent symptoms. It is important to remember that a dermoid cyst might also be found by chance. Unexpected headaches, convulsions, or more serious problems such as chemical meningitis, vasospasm, and cerebral infarction may also result from cyst ruptures [3].

Because of its multiplanar format and excellent spatial resolution, MRI is the preferred diagnostic method for accurately identifying the site of dermoid tumours and the involvement of nearby tissues. It provides clear evidence of the fat components that are typical of dermoid tumours. Magnetic resonance imaging is also more effective at detecting fat droplets caused by the rupture of a dermoid tumour that is located in the ventricles or subarachnoid regions [4]. Dermoid tumours can be found for the first time on CT scans. Low attenuation readings that are consistent with fat are suggestive of a dermoid tumour diagnosis. Dermoid tumours frequently contain calcifications, which are best detected using a CT scan [5]. Ultrasonography has been used to evaluate subgaleal dermoid cysts of the anterior fontanelle in young children, despite its limited utility in the evaluation of central nervous system (CNS) dermoid malignancies. Because a dermoid tumour appears on angiography as an avascular mass, dermoid and epidermoid tumours are rarely studied using angiography. However, angiography may effectively show the displacement or encasement of blood vessels close to the tumour, i.e., magnetic resonance angiography (MRA) or computed tomography angiography (CTA). Fat signal intensity in the ventricles or subarachnoid space indicates the rupture of dermoid cysts. Some fluid-attenuated inversion recovery (FLAIR) sequences may be "fat suppressed" by nature, which reduces the signal from the globules of fat in the subarachnoid space. If chemical meningitis complicates a ruptured dermoid cyst, leptomeningeal response and enhancement can be seen [6].

It is crucial to take into account any additional cerebral diseases and chemicals that might have contributed to the T1 high signal while monitoring one. These include the byproducts of haemoglobin breakdown, protein-rich compounds, and melanin-containing tumours like melanoma and colloid cysts. It is important to consider other possible diagnoses when making a differential diagnosis for lesions that contain minerals, including calcium, copper, and manganese. However, the apparent "blooming artefact" that is present in the dermoid and subarachnoid fat droplets makes the use of T2*-weighted sequences in imaging a substantial disadvantage. The presence of hemosiderin staining or a ruptured dermoid cyst may be mistaken for a rapid subarachnoid haemorrhage in these regions, which present as regions of low signal [7].

Treatment for a ruptured intracranial dermoid cyst

Conservative management can also be done if the dermoid is not ruptured and is not causing any mass effect. If it is symptomatic, endonasal decompression can be done. Another surgical approach is a craniotomy, with the evacuation of the lesion and irrigation of subarachnoid spaces for the removal of ruptured contents and debris. Recurrence is not so common, but if the capsule of the dermoid is not excised completely, there might be a recurrence, but it's still very rare.

## Conclusions

An intracranial dermoid cyst rupture is a rather uncommon occurrence. When such a rupture occurs, patients frequently have headaches, seizures, and other indications that are caused by the compression of the eyes and nearby structures. Radiological characteristics and the presence of dispersed fat droplets in the subarachnoid space or ventricles can be used to make the diagnosis of a burst dermoid cyst. It's crucial to remember that headaches can have a variety of reasons, and it is uncommon for dermoid cysts, or the ones that burst to induce chemical meningitis or headache and eye symptoms. The importance of determining the underlying cause of headaches and eye problems is highlighted by this case report, with MRI serving as the go-to diagnostic technique.

## References

[1] Muçaj S, Ugurel MS, Dedushi K, Ramadani N, Jerliu N (2017). Role of MRI in diagnosis of ruptured intracranial dermoid cyst. Acta Inform Med.

[2] Osborn AG, Preece MT (2006). Intracranial cysts: radiologic-pathologic correlation and imaging approach. Radiology.

[3] Cohen JE, Abdallah JA, Garrote M (1997). Massive rupture of suprasellar dermoid cyst into ventricles. Case illustration. J Neurosurg.

[4] Brown JY, Morokoff AP, Mitchell PJ, Gonzales MF (2001). Unusual imaging appearance of an intracranial dermoid cyst. AJNR Am J Neuroradiol.

[5] Stephenson TF, Spitzer RM (1987). MR and CT appearance of ruptured intracranial dermoid tumors. Comput Radiol.

[6] Venkatesh SK, Phadke RV, Trivedi P, Bannerji D (2002). Asymptomatic spontaneous rupture of suprasellar dermoid cyst: a case report. Neurol India.

[7] Zimny A, Zińska L, Bladowska J, Neska-Matuszewska M, Sąsiadek M (2013). Intracranial lesions with high signal intensity on T1-weighted MR images - review of pathologies. Pol J Radiol.

